# Supplementary material for: Cytotoxicity Comparison of 99mTc-Labeled Peptide Antagonist and Agonist Targeting the SSTR2 Receptor in AR42J Cells
Source: Molecules. 2025 Apr 11;30(8):1715. doi: 10.3390/molecules30081715 (PMC12029662; doi:10.3390/molecules30081715)
Supplement: Supplementary file 1 [file molecules-30-01715-s001.zip › molecules-3520092-supplementary.pdf]

molecules

MDPI

Supplementary Materials

Cytotoxicity Comparison of ^{99m}Tc-Labeled Peptide Antagonist and Agonist Targeting the SSTR2 Receptor in AR42J Cells

Sahar Nosrati Shanjani [1], Monika Łyczko [1], Rafał Walczak [1], Przemysław Koźmiński [1], Emilia Majka [1], Jerzy Narbutt [1], Wioletta Wojdowska [2], Agnieszka Majkowska-Pilip [1,*] and Aleksander Bilewicz [1,*]

1 Institute of Nuclear Chemistry and Technology, Dorodna 16, 03-195 Warsaw, Poland; sahar.nosrati.shanjani@gmail.com (S.N.S.); m.lyczko@ichtj.waw.pl (M.Ł.); r.walczak@ichtj.waw.pl (R.W.); p.kozminski@ichtj.waw.pl (P.K.); e.amajka@ichtj.waw.pl (E.M.); j.narbut@ichtj.waw.pl (J.N.)
2 National Centre for Nuclear Research, Sołtana 7/3, 05-400 Otwock, Poland; wioletta.wojdowska@polatom.pl
* Correspondence: a.majkowska@ichtj.waw.pl (A.M.-P.); a.bilewicz@ichtj.waw.pl (A.B.)

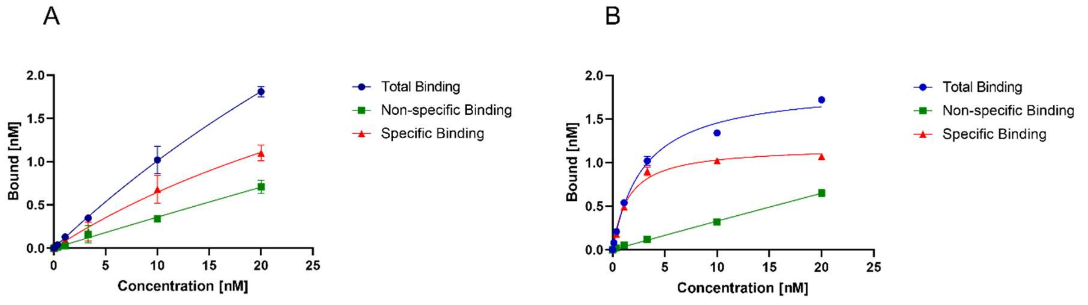

Figure S1. Saturation binding assay with ^{99m}Tc-labeled agonist (A) and antagonist (B) for determining the binding affinity in somatostatin overexpressing AR42J cell line. Shown are the total binding, specific binding, and non-specific binding in the presence of a 2000-fold excess of octreotide.

Table S1. K_D and B_{max} values determined from saturation curves for ^{99m}Tc-TEKTROTYD and ^{99m}Tc-TECANT-1.

Radiobioconjugate	K_D [nM]	B_{max} [nM]
^{99m}Tc-TEKTROTYD	- *	3.9
^{99m}Tc-TECANT-1	1.5	1.2

* Because of the lack of specific binding curve saturation, the K_D value could not be reliably determined..

 https://doi.org/10.3390/molecules30081715
